# Biomarkers in Rare Diseases 2.0

**DOI:** 10.3390/ijms23094582

**Published:** 2022-04-21

**Authors:** Bridget E. Bax

**Affiliations:** Molecular and Clinical Sciences Research Institute, St. George’s, University of London, London SW17 0RE, UK; bebax@sgul.ac.uk

It is estimated that there are over 7000 rare diseases, collectively affecting more than 350 million individuals worldwide. Despite rare disease drug development representing one of the fastest-growing areas for pharmaceutical research and development investment and the incentives offered through national orphan drug designation initiatives, less than five percent of rare diseases have licenced treatments. Rare diseases are a global challenge; the absence of natural history data, limited disease pathophysiology knowledge, disease heterogeneity and small patient numbers have a discordant impact on the rare disease drug development process.

The identification of dynamic biomarkers such as gene expression, metabolites, inflammatory markers and proteins have become increasingly important tools for overcoming some of the above challenges. Increasingly, rare disease drug developers are undertaking biomarker strategies and applying high throughput platforms to evaluate exploratory biomarkers. These activities have led to significant advances in some rare disease drug development programmes through utilizing biomarkers as secondary or exploratory endpoints measures in clinical trials and stratifying patients according to disease subtype. Without doubt, rare disease biomarker discovery is an active research field, but much more work is needed to expand the portfolio of biomarkers for those rare diseases with unmet needs.

This Special Issue entitled “Biomarkers in Rare Diseases 2.0” is a continuation of the first Special Issue. The objective is to provide a platform for original research articles and state-of-the-art reviews on novel or established molecular biomarkers that contribute to the understanding of the underlying molecular mechanisms of rare diseases and/or that can be used for the diagnosis and prognosis of disease and individuals’ responses to therapies. The collection includes four research articles and eight reviews involving a total of 87 different contributors.

The original article of Steponaitis and Tamasauskas provides an excellent example of using a biomarker signature to stratify disease subtypes [1]. Through the selection of a 30-gene expression signature for glioma stem cells, the authors demonstrated that glioblastomas of proneural and mesenchymal subtypes could be partitioned into different clusters. The molecular partitioning of glioblastoma subtypes was also found to be significantly linked to patient outcome, demonstrating that clinical outcome may be determined by distinct glioma stem cell populations. Further development of these finding could potentially lead to personalized therapeutic strategies.

Two original articles in this Special Issue focused on biomarkers for mitochondrial disorders. Due to their clinical and genetic heterogeneity, mitochondrial disorders are extremely challenging to diagnose. Although the cytokines fibroblast-growth factor 21 (FGF-21) and growth-differentiation factor 15 (GDF-15) are considered to be favourable indicators of mitochondrial disease and particularly for those with muscle involvement, these cytokines are also often associated with a variety of non-mitochondrial pathologies. The complexity of these disorders is also likely to preclude the identification of a single relevant biomarker and thus the identification of a biomarker panel may be more appropriate. In their eloquent study, Peñas and co-workers, investigated the diagnostic performance of the plasma isoform of gelsolin (pGSN) relative to FGF-21 and GDF-15 [2]. Their focus on GSN was based on previous proteomics-based studies showing this protein to be a potential therapeutic target for OXPHOS dysfunction. A combination of pGSN and GDF-15 was shown to preferentially discriminate between mitochondrial disease and non- mitochondrial disease for subjects under the age of 50 years, whereas FGF-21 best classified older subjects. The authors concluded that pGSN could improve the diagnostic capacity of GDF-15 and FGF-21 when applied to this restricted age group.

By definition, patients with rare diseases are few in number and thus, geographically scattered. The development of global academic collaborations is pivotal to the advancement of knowledge, particularly for diseases which are classified as ultra-rare. Organizations such as the International Rare Diseases Research Consortium (IRDiRC), Rare Diseases International and RareConnect actively promote international collaboration for the advancement of rare diseases research worldwide. The second article focusing on mitochondrial disease is an excellent example of an international collaborative effort between ten countries and 20 collaborators. In their four-phase study, Mencias and co-workers investigated the utility of microRNAs (miRNAs) as potential circulating biomarkers of the ultra-rare disease, mitochondrial neurogastrointestinal encephalomyopathy (MNGIE) [3]. Of the five plasma miRNAs and three serum miRNAs that could robustly distinguish MNGIE disease from healthy controls, the single best predictor was miR-34a-5p. A decrease in the expression of miR-34a-5p was noted in four patients treated with erythrocyte-encapsulated thymidine phosphorylase (EE-TP), an enzyme replacement therapy under clinical development for MNGIE, thus demonstrating the potential utility of this miRNA in monitoring response to treatment. The authors concluded that the plasma exploratory miRNA biomarker panel could be of prognostic value in assessing clinical status and should be included in future clinical trials of investigational therapies for MNGIE.

The fourth original article is that of Basile and colleagues who investigated the diagnostic-prognostic biomarker potential of hybrid IgG4 k/λ antibodies in patients with a specific disease subtype of Myasthenia gravis [4]. The disease subtypes are classified according to the production of antibodies against the muscle-specific tyrosine kinase (MuSK) or the acetylcholine receptor (AChR), with the former having a proven IgG4-mediated pathogenicity. IgG4 molecules stochastically exchange half-molecules with other IgG4s, referred to as Fab-arm exchange. The study demonstrated that in contrast with the AChR subtype, the MuSK subtype had a significant correlation between the hybrid/ total IgG4 ratio and anti-MuSK antibody titres. In addition, an increase in the mean hybrid/total IgG4 ratio was shown to correlate with disease severity. The potential value of hybrid IgG4 molecules as diagnostic-prognostic biomarkers for MuSK- myasthenia gravis warrants further investigation.

Two contributions in this Special Issue focus on biomarkers of autoimmune disorders. Ciano-Petersen and colleagues provide an important overview of the biomarkers described to date in anti-N-methyl-D-aspartate receptor (NMDAR) encephalitis [5]. The authors highlight that despite the large efforts that have been made to discover soluble molecules since the disease was first identified in 2007, a majority of these biomarkers still remain at the exploratory phase. A notable exception, however, is the cerebrospinal fluid IgG NMDAR antibodies that are now exploited in clinical practice. The authors recognise the need to decipher the pathophysiology of this disorder and that international collaborative studies would be required to increase patient recruitment and achieve statistical power. The review of Maciak and co-workers, give special attention to Th17 cells and Th17-related cytokines in the context of their potential usefulness as discriminatory markers for the two distinct autoimmune inflammatory diseases, multiple sclerosis and neuromyelitis optica [6]. The two disorders present similar clinical features and thus an improved understanding of the immunopathogenic mechanisms of each disease is essential to enhance their discriminatory diagnosis. Findings indicate that factors specific to the Th17 pathway permit differentiation between the two disorders and healthy individuals, with IL-6 being particularly important. The authors conclude that in depth studies of Th17-related pathways may lead to the availability of more effective therapies. 

Fotiou and colleagues present a comprehensive review of 112 publications that focus on biomarkers of immunoglobulin light chain (AL) amyloidosis, a rare and heterogenous haematological disease [7]. The disorder is characterized by free immunoglobulin light chain misfolding and amyloid deposition on target tissues, leading to organ dysfunction. The authors argue that sensitive biomarkers are urgently needed due to current staging systems falling short of their prognostic ability. Although a number of new biomarkers have been reported in recent years, none have been incorporated as exploratory endpoints in clinical trials or clinical practice. The authors surmise that it is unlikely that a single biomarker would be specific enough to reflect the complexity and heterogeneity of the disease.

The contribution of Caliogna and co-workers provides an interesting overview of Ehlers-Danlos syndromes (EDS), an inherited heterogeneous group of connective tissue disorders which are characterized by an abnormal collagen synthesis affecting skin, ligaments, joints, blood vessels, and other organs [8]. Their review of 93 articles focused on identifying possible biomarkers for the two most common forms of EDS, the classic and the hypermobile. The clinical and genetic heterogeneity of EDS makes it difficult to diagnose patients and additionally the disease has many features that are common to other heritable connective tissue disorders. The authors concluded that EDS disease aetiology is poorly researched and recommend investigation into new potential biomarkers that would confirm patients’ diagnoses and disease progression.

Molares-Vila and colleagues present the first ever review of emerging biomarkers for inherited glycogen storage diseases (GSDs), a group of 19 diseases [9]. GSD are caused by a deficiency in one or more enzymes involved in the synthesis or degradation of glycogen and are characterized by deposits or abnormal types of glycogen in tissues. After reviewing 145 papers published between 1997 and 2020, the authors deduced that a majority of the identified biomarkers required clinical validation, with only calprotectin for hepatic GSDs and urinary glucose tetrasaccharide for Pompe disease having achieved approval for clinical use. The authors are of the opinion that research into biomarkers of GSD is over-shadowed by the current focus on gene therapy and hope that the biomarkers described in their review will open up new horizons for improving diagnosis, prognosis and therapeutic approaches.

The interesting review of Li and Chen provides a comprehensive summary of studies that have elucidated the role of the three endoplasmic reticulum (ER) stress signalling pathways in the pathogenesis of kidney disease, specifically, inositol-requiring enzyme 1, protein kinase R-like ER kinase and activating transcription factor 6 signalling [10]. The authors highlight the recently identified endoplasmic reticulum associated biomarkers MANF, ERdj3, ERdj4, CRELD2, PDIA3, and angiogenin and recommend the implementation of these to enhance the understanding of rare kidney disease pathogenesis and provide tools for early diagnosis, risk stratification and treatment response monitoring.

The review of Mrozikiewicz and co-authors summarizes the pathology of recurrent implantation failure (RIF), an important problem and an enormous challenge for human reproductive medicine which is often overlooked in the context of rare diseases [11]. RIP is defined as three or more consecutive failed in vitro attempts with at least four high-quality embryos in a minimum of three fresh or frozen cycles. It is estimated that 5% of women suffer from recurrent pregnancy loss (RPL), of which 75% of cases are diagnosed as RIF. There is thus a great need for diagnostic tests that could provide assessments of RIF and RPL risk. From their review of 119 papers, the authors conclude that associations exist between RIP occurrence and wide range of variables, including hormones, angiogenic and immunomodulatory factors and genetic polymorphisms. Further research is required to identify RIP biomarkers that could potentially provide individualized treatment plans and ultimately improve the chance of pregnancy.

The compelling review of Kang and co-workers investigated studies that reported biomarkers related to chronic subjective tinnitus [12]. Tinnitus leads to a poor quality of life by inducing sleep disorders and psychiatric distress. The pathophysiology of chronic subjective tinnitus is not clearly elucidated and currently there are no objective markers that can indicate diagnosis, evaluation, and the effectiveness of treatment. The authors applied search terms “Tinnitus”, “Biomarker” and “Marker”, to identify 619 articles in three databases. After implementing a set of exclusion criteria, 49 studies remained, these being the subject of this review. A total of 58 biomarkers were identified as indicators for diagnosis, evaluation, prognosis, and therapeutic effectiveness of tinnitus, and were classified into metabolic, haemostatic, inflammatory, endocrine, immunological, neurologic, and oxidative parameters. The authors recommend that an individualized and diversified approach should be applied to the diagnosis and treatment of tinnitus, rather than employing a standardized methodology.

The studies and reviews reported in this Special Issue reveal the diversity of biomarkers that have potential application to the management of a wide range of rare diseases. We hope these findings will lead to new avenues of research that will unlock the current challenges that face rare diseases and ultimately shorten rare disease drug development timelines.
